# Androgen receptor expression in glioblastoma: molecular profiling and association with tumor burden

**DOI:** 10.1007/s11033-026-11673-6

**Published:** 2026-03-24

**Authors:** Larysa Liubych, Lorenz Hahn, Raja Hollnagel, Maria Karolin Streubel, Deepak Ailani, Jan Leppert, Harald Krenzlin, Jakob Matschke, Celina Soltwedel, Nicole Borgardt, Susanne Behling, Oksana Zemskova, Claudia Ditz, Niklas Gebauer, Maxim Kebenko, Naureen Keric, Olga Lapshyna, Timo Gemoll, Cedric Carl, Alexander Neumann, Dirk Rades, Anastassia Löser

**Affiliations:** 1 Department of Radiotherapy, University Medical Center Schleswig-Holstein, Lübeck, Germany; 2 Department of Neurosurgery, Laboratory for Experimental Neuro-Oncology, University Medical Center Schleswig-Holstein, Lübeck, Germany; 3 Department of Neurosurgery, University Medical Center Schleswig-Holstein, Lübeck, Germany; 4 Institute of Neuropathology, University Medical Center Hamburg-Eppendorf, Hamburg, Germany; 5 State Institution Romodanov Neurosurgery Institute, National Academy of Medical Sciences of Ukraine, Kyiv, Ukraine; 6 Department of Hematology and Oncology, University Medical Center Schleswig-Holstein, Lübeck, Germany; 7 Department of Surgery, Laboratory of Surgical Research, University Medical Center Schleswig-Holstein, Lübeck, Germany; 8 Strahlentherapie Nord, Center for Radiotherapy, Bremen Gröpelingen, Germany; 9 Department of Neuroradiology, University Medical Center Schleswig-Holstein, Lübeck, Germany; 10 Department of Radiotherapy, University Medical Center Schleswig-Holstein Campus Lübeck, Ratzeburger Allee 160, 23562 Lübeck, Germany

**Keywords:** Glioblastoma, Androgen receptor, mRNA, Protein expression, Tumor volume

## Abstract

**Background:**

Glioblastoma (GBM) is the most aggressive primary brain tumor in adults, characterized by higher incidence and poorer outcomes in males. Increasing evidence suggests that androgen receptor (AR) expression may influence GBM progression, yet its clinical significance remains uncertain. This study aimed to evaluate AR expression at both mRNA and protein levels in GBM tissue and to explore its potential association with magnetic resonance imaging (MRI)-defined tumor volume.

**Methods and results:**

Tumor samples from 34 patients with primary GBM were analyzed. AR mRNA expression was quantified by real-time PCR in 30 cases, while immunohistochemistry was performed in 24 cases to assess AR protein expression. Tumor volumes were determined from T1-contrast-enhancing and FLAIR-hyperintense MRI regions. Statistical analyses included unpaired t-tests with Welch’s corrections and Spearman’s rank correlations with additional multivariable linear regression analyses (pre-steroid imaging status was not included). Mean AR mRNA and protein expression levels were higher in males than females, though not statistically significant. AR mRNA expression showed a strong trend toward positive correlation with Ki67 proliferation index (*r* = 0.44, *p* = 0.07) and tumor volume (*r* = 0.36, *p* = 0.06 for T1-enhancing regions; *r* = 0.40, *p* = 0.03 for non-enhancing FLAIR-hyperintense regions). Exploratory multivariable model adjusted for age, sex, and MGMT status revealed that higher AR mRNA expression was independently associated with FLAIR-hyperintense tumor volume (β = 0.50, *p* = 0.037), while AR protein expression and all other covariates showed no significant associations.

**Conclusion:**

AR expression is consistently detectable in GBM tissue and shows a trend toward association at the transcriptional level with non-contrast-enhancing tumor volume, suggesting a potential role in GBM biology and warranting further investigation in larger studies.

**Supplementary Information:**

The online version contains supplementary material available at 10.1007/s11033-026-11673-6.

## Introduction

Glioblastoma (GBM) is the most aggressive and lethal form of malignant glioma [1]. According to the fifth edition of the WHO Classification of Tumors of the Central Nervous System in 2021, it is characterized by its isocitrate dehydrogenase (IDH) wildtype and its infiltrative nature and resistance to conventional therapeutic interventions [1, 2]. The primary treatment options for GBM include surgical resection, followed by combined chemoradiotherapy and maintenance chemotherapy (e.g. with DNA-alkylating temozolomide) ± tumor treating fields [2–4].

Epidemiologically, GBM exhibits a pronounced sex disparity [5, 6]. While the incidence of low-grade gliomas is similar among both genders, the incidence of higher-grade gliomas (including GBMs) clearly predominates in males with shorter overall survival and poorer outcome [7–9]. GBMs present the highest incidence of primary malignant tumors of the central nervous system (48.6%) [10, 11] with an incidence rate in male patients which is 1.6 times higher than in female patients [7, 12, 13].

One emerging avenue of exploration involves understanding the role of androgen receptor (AR) expression in GBM. Traditionally, the effects of male sex hormones such as testosterone and dihydrotestosterone are mediated via the AR, a steroid hormone receptor [14]. Recently, AR have garnered attention beyond their classical roles, which typically included the development of the male reproductive system and secondary sexual characteristics [14]. Since the 1990s, studies have revealed the presence of ARs in various non-reproductive tissues, including the brain [15, 16]. AR, encoded by the *NR3C4* gene (nuclear receptor subfamily 3, group C, member 4), is a nuclear receptor and key transcription factor that mediates androgen-induced signaling. It functions as a critical effector molecule in numerous physiological and pathophysiological processes. In its inactive state, AR resides in the cytoplasm bound to heat shock proteins. Upon ligand binding, the receptor undergoes conformational changes, dissociates from the chaperone complex, and translocates into the nucleus, where it regulates the transcription of androgen-responsive genes [17].

In the context of GBM, the expression and functional significance of AR have become a subject of intense research [18–24]. Evidence suggests that AR expression correlates with glioma malignancy [7]. AR has been reported in 37.5% of GBMs, 13.3% of anaplastic astrocytomas and 8.3% of astrocytomas [15, 25–27]. Among all three glioma types, GBMs showed the highest AR expression [7, 28]. Tumor tissue samples from GBM patients reveal increased AR expression compared to peripheral normal brain tissue. AR expression has also been detected in the human GBM cell lines U87MG, A172, LN18, LN229, M059, T98G, U118MG, and U138MG [7, 28–30].

AR activation, either in an androgen-dependent or independent manner, promotes GBM cell proliferation and plays a critical role in its progression with possible involving TGF-β/LIF/STAT3 signaling pathways [31–36]. Preclinical models targeting GBM with AR antagonists (e.g., enzalutamide or seviteronel) demonstrate promising results, inducing cell death, increasing radio- and chemosensitivity and reducing tumor volume [18, 20, 37–44].

AR expression in GBM is higher in males than in females but shows no statistically significant association with patient sex [21, 22, 30, 40], demonstrating the need for continued research.

While the role of the AR in tumorigenesis is well characterized in malignancies such as prostate cancer, its contribution to the development and progression of GBM, as well as its impact on gene expression in GBM cells, remains poorly understood [7, 18–20]. This analysis aims to examine the potential correlation between AR expression in GBM tissue and tumor volume, thereby providing insights into the possible role of AR in GBM pathophysiology. Since AR expression in GBM has been reported, the unique contribution of this study and the main research question is whether AR expression in GBM is associated with magnetic resonance imaging (MRI)-defined tumor volume.

## Materials and methods

### Ethical approval and patient consent

This study was conducted in compliance with the Declaration of Helsinki and all relevant national and international regulations. The use of human biological material was approved by the Ethics Committee of the University of Lübeck, Germany (approval number: 2024 − 219). As most patients had already deceased due to the rapid progression of the disease, obtaining renewed written consent was not feasible. However, all patients had previously consented to the use of their clinical data and biological specimens for research purposes as part of the general hospital documentation. The use of archival samples from deceased patients was explicitly covered by ethics approval.

### Patient samples and tissue collection

**Fig. 1:**
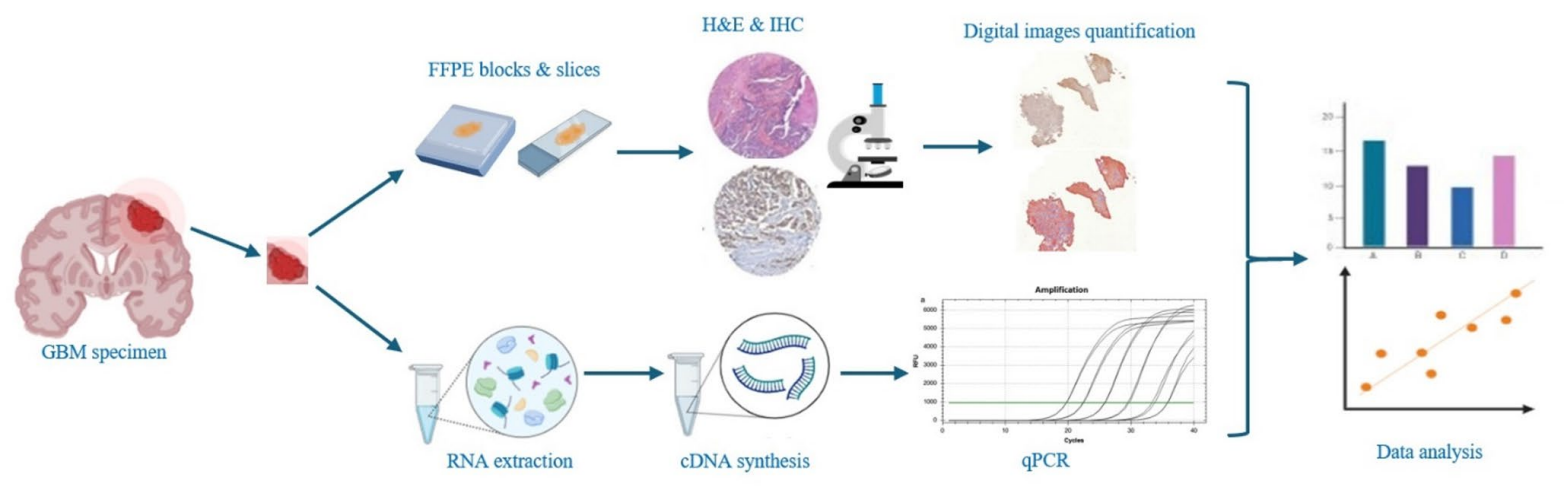
Scheme of the applied methods. cDNA – complementary deoxyribonucleic acid; FFPE – formalin fixed & paraffin-embedded tissue samples; GBM – glioblastoma; H&E – hematoxylin-eosin staining; IHC – immunohistochemical staining; RNA – ribonucleic acid; qPCR – quantitative real-time polymerase chain reaction

Tumor material was obtained from 34 patients undergoing surgical resection for histopathologically confirmed GBM, diagnosed in accordance with current treatment guidelines. All tumors were IDH-wildtype. The analyzed specimens originated from the enhancing tumor region adjacent to the tumor core, as identified by preoperative neuroimaging (MRI). Surgical procedures included standard craniectomy followed by tumor resection. A schematic overview of the workflow is presented in Fig. 1, which was created using the free version of BioRender.com.

### Quantitative real-time PCR (qPCR)

Fresh-frozen tumor samples from 30 primary GBM patients were processed for RNA extraction using TRIzol™ Reagent (Thermo Fisher Scientific), followed by DNase I (RNase-free, Thermo Fisher Scientific) treatment. RNA purity and concentration were assessed using a Nanodrop ND–1000 spectrophotometer (Thermo Fisher Scientific). Complementary DNA (cDNA) was synthesized from 1 µg of total RNA per sample using the iScript™ cDNA Synthesis Kit (Bio-Rad).

qPCR was performed using the SYBR Green qPCR Master Mix (Thermo Fisher Scientific) on a real-time PCR system. AR-specific primers [30], validated using the Basic Local Alignment Search Tool (BLAST) [45], were: forward: 5’-CCAGGGACCATGTTTTGCC-3’; reverse: 5’-CGAAGACGACAAGATGGACAA-3’. Glyceraldehyde-3-phosphate dehydrogenase (GAPDH) served as the reference gene, with primer sequences: forward: 5’-GGAGCGAGATCCCTCCAAAAT-3’; reverse: 5’-GGCTGTTGTCATACTTCTCATGG-3’.

Thermal cycling conditions included an initial denaturation at 95 °C for 15 s, followed by 40 cycles of 95 °C for 15 s and 60 °C for 60 s. Effective concentrations of primers and cDNA were estimated in previous experiments. Each sample was analyzed in triplicate, and mean Ct values (threshold cycle) were used for calculations (assuming a single confirmed peak for the melting curve, Suppl. Figure 1). AR mRNA expression was normalized to GAPDH and quantified using internal calibrator control sample and ΔΔCt method, with results expressed as 2^(−ΔΔCt)^ [46]. A cDNA sample obtained from one of the tumor GBM samples and previously calibrated in experiments with cDNAs obtained from primary cultures of GBM stem cells (positive control) was used as an internal calibrator control sample.

### Immunohistochemistry (IHC)

Paraffin-embedded tumor specimens from 24 patients were prepared from tissue fixed in 4% formaldehyde for 24 h. Serial sections (4 μm) were cut using a microtome (Microm HM340E, Thermo Fisher Scientific) and mounted on poly-L-lysine-coated glass slides (Superfrost™ Plus Adhesion Microscope Slides, Epredia).

Sections were deparaffinized, rehydrated, and subjected to antigen retrieval in 10 mM citrate buffer (pH 6.0) by microwaving at 900 W for 3 min followed by 600 W for 8 min (twice). After cooling and PBS washing, permeabilization was performed with 0.1% Triton X-100 in PBS for 10 min. Endogenous peroxidase activity was blocked with 3% H₂O₂ in methanol for 10 min. Non-specific binding was blocked with normal horse serum (1:50) for 25 min.

Sections were incubated overnight at 4 °C with mouse anti-human AR monoclonal antibody (sc-7305, 1:200, Santa Cruz Biotechnology) or mouse anti-human Ki67 antigen antibody (clone MiB-1, 1:200, DaKo, Denmark). The negative control for each GBM tissue sample were parallel sections of the same GBM tissue with the primary antibody step removed.

Detection was performed using the VECTASTAIN Elite ABC-HRP kit (Vector Laboratories) with biotinylated secondary horse anti-mouse/rabbit antibody, followed by streptavidin–horseradish peroxidase and visualization with diaminobenzidine (DAB). Nuclei were counterstained with hematoxylin, and sections were mounted using Aquatex medium (Merck KGaA) with glass coverslips (Th. Geyer GmbH & Co. KG).

Digital images were acquired using a 3DHISTECH Panoramic Desk scanner and analyzed with Pannoramic Viewer (version 1.15.4). For each sample, the same enhancing region was analyzed on all sections, excluding areas of necrosis/vessels. Quantification of AR-positive cells was performed automatically using QuPath software (version 0.5.1), based on DAB-positive nuclei detection [41] (score compartment “Nucleus: DAB optical density (OD) mean”) as well as DAB-positive cytoplasm+nuclei detection (score compartment “Cell: DAB OD mean”). Quantification of Ki67 positive cells was performed based on DAB-positive nuclei detection. The following thresholding criteria were defined: resolution – moderate (2.62 μm/px); channel – hematoxylin; prefilter – weighted deviation; smoothing sigma – 2; threshold – 0.01. The median analyzed area was 10,000 µm^2^.

Results were expressed as the percentage of positive cells relative to the total number of cells within the analyzed area of enhancing tumor region.

### Tumor volume assessment

Preoperative magnetic resonance imaging (MRI) was used to measure tumor volumes. T1-contrast-enhancing regions were analyzed for initial tumor volume (baseline), and FLAIR-hyperintense regions were evaluated for T1-negative components. Tumor segmentation was performed using the Brainlab^®^ software (Munich, Germany) employing a semi-automated workflow. Initial tumor contours were generated automatically on contrast-enhanced T1-weighted MRI scans and subsequently reviewed and manually refined by experienced clinicians. Necrotic tumor components and peritumoral edema were excluded from volumetric analysis and were identified and delineated manually to ensure consistency across cases. The persons performing the assessment were blinded to the AR data.

MRI scans used for tumor volumetry were acquired at baseline; however, information on prior steroid administration was not consistently available for all patients, as a subset of cases was referred from external institutions. Consequently, while imaging was performed as close as possible to initial diagnosis, a uniform pre-steroid imaging status cannot be unequivocally confirmed for all patients.

### Statistical analysis

Data analysis was conducted using IBM SPSS Statistics version 27 (IBM Corp., 2020). Graphical representations were created in GraphPad Prism version 10.0.0 (GraphPad Software). Normality was assessed using the Shapiro–Wilk and D’Agostino & Pearson tests. For comparisons between independent groups (*n* = 2 in each comparison), the unpaired t-test with Welch’s correction was applied. Additionally the effect sizes (Cohen’s d) and 95% confidence intervals (CI) were calculated.

Sex (male vs. female) and age (< 60 vs. ≥60 years) were included as stratification factors, in accordance with established clinical research and WHO classification criteria [47–49]. Correlations were evaluated using Spearman’s rank correlation coefficient. Since the analyzed indicators were continuous values, data are reported as means with 95% CI: mean (95% CI [lower CI; upper CI]). Statistical significance was defined as *p* < 0.05.

An exploratory multivariable linear regression models adjusted for age, sex, and O^6^-methylguanine-DNA methyltransferase (MGMT) status were performed in R environment (R version 4.5.2, 2025-10-31 ucrt). Separate models were fitted with tumor volume (T1-contrast-enhancing or FLAIR-hyperintense regions) as the outcome, and AR expression together with age, sex, MGMT status and proliferation (% Ki67 positive cells) as predictors. Tumor volumes were log-transformed prior to analysis to reduce skewness. Results are reported as the regression coefficient β wit 95% CI; statistical significance was defined as *p* < 0.05.

Pre-steroid imaging status was not included in the multivariable model because this information was not consistently available for all patients, and excluding cases with unknown steroid use would have markedly reduced the sample size.

## Results

### Patient characteristics

The study cohort consisted of 34 patients with primary GBM, with an almost equal sex distribution (18 males, 16 females). The median age was 70 years (range 46–88 years), with 11.8% of patients (*n* = 4) younger than 60 years and 88.2% older than 60 years (*n* = 30). 21 of 34 patients (61.8%; 13 males, 8 females) had negative MGMT promoter methylation status, 13 (38.2%; 5 males, 8 females) – MGMT-positive status. Epidermal growth factor receptor (EGFR) - amplification status was not available in majority of cases, therefore not provided.

Patient characteristics are summarized in Table 1.

**Table 1:** Baseline patient characteristics

Sample size	Feature	Index
	Gender: male / female	18 (52.9%) / 16 (47.1%)
	Age (years; at initial diagnosis)	70 (range 46-88)
	MGMT-status: negative / positive	21 (61.8%) /13 (38.2%)
	Human AR mRNA expression (qPCR)	0.76 (95% CI [0.53; 0.99])
	Percentage of AR-positive cells (IHC staining)	nuclei: 20.49 (95% CI [13.35; 27.63])%
		cells: 32.33 (95% CI [22.53; 42.12])%
	Percentage of Ki67-positive cells (IHC staining)	27.17 (95% CI [18.96; 35.39])%
	Tumor volume on post-contrast T1-weighted MRI (cm³)	36.22 (95% CI [26.14; 46.30])
	Tumor volume on T2-weighted FLAIR MRI (cm³)	90.91 (95% CI [74.05; 107.77])

All patients presented with primary glioblastoma

AR – androgen receptor; qPCR – quantitative polymerase-chain reaction; IHC – immunohistochemistry; MGMT – O^6^-methylguanine-DNA methyltransferase; MRI – magnetic resonance imaging; FLAIR – fluid-attenuated inversion recovery

### Tumor volume

**Fig. 2:**
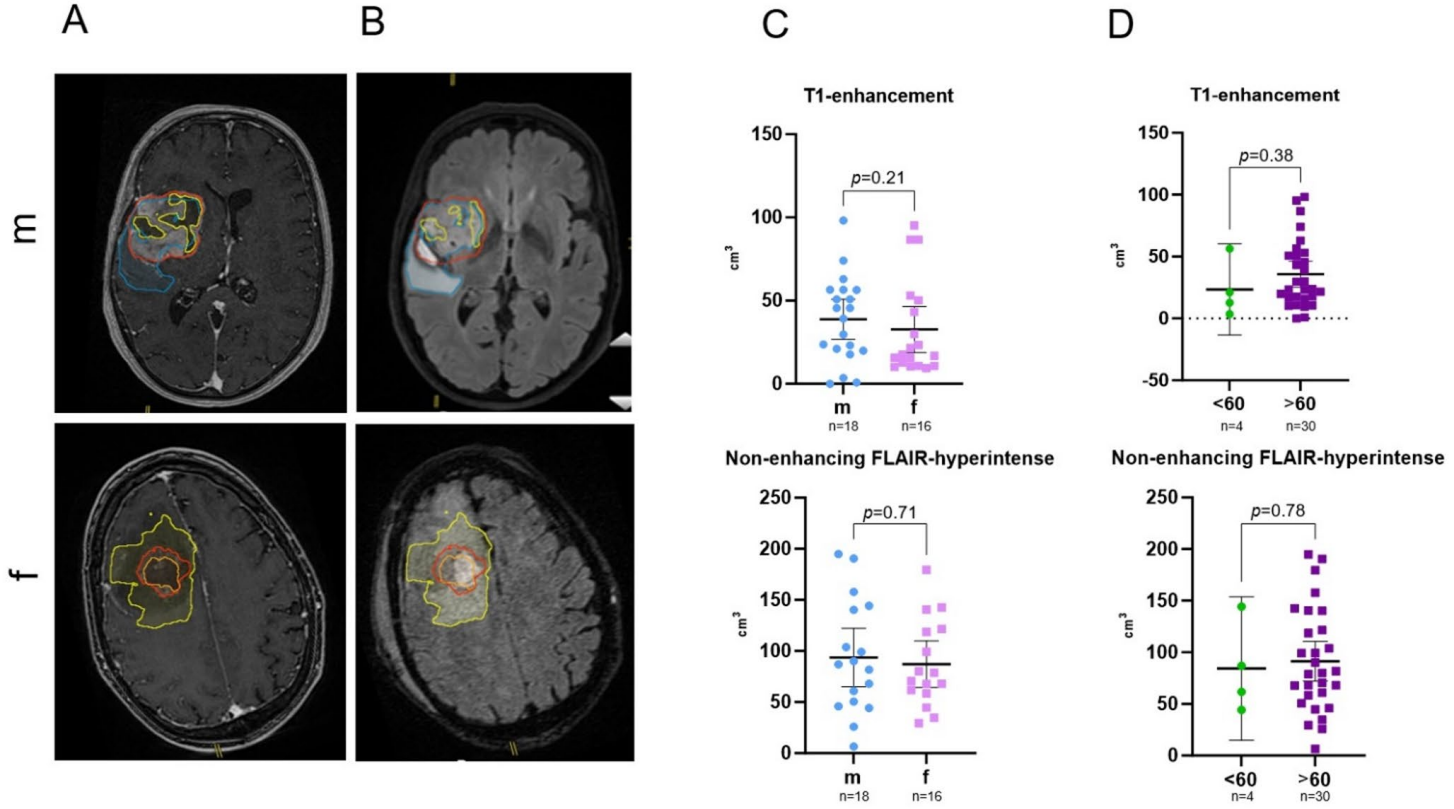
Tumor volume in glioblastoma (GBM) patients. Representative axial images of T1-contrast-enhanced MRI (**A**) and T2 FLAIR-hyperintense regions (**B**) (red – contrast-medium uptake; blue – perifocal edema; yellow – necrotic area) made on different days (8 days apart) from the same male (m) and female (f) patients. Scatter dot plots showing mean with 95% CI for initial tumor volume (T1-enhancement & T2 FLAIR-hyperintense regions) in GBM patients depending on sex (**C**) and age (**D**). m – male, f – female; <60 – patients younger than 60 years, > 60 – patients older than 60 years. FLAIR – fluid-attenuated inversion recovery

The average initial tumor volume, assessed by T1-contrast-enhanced MRI (Fig. 2, A**)**, was 36.22 cm³ (95% CI [26.14; 46.30] cm³). When stratified by sex, males showed a mean volume of 40.24 cm³ (95% CI [25.05; 55.43] cm³) compared to 31.69 cm³ (95% CI [17.23; 46.15] cm³) in females (*p* = 0.21, see Fig. 2, C). Small effect size (Cohen’s d = 0.18) with wide CI (95% CI [–0.50; 0.87]) confirmed the absence of a statistically significant difference between groups (see Supplementary table).

The volume of T1-negative, non-enhancing FLAIR-hyperintense regions (Fig. 2, B) was greater, with an average of 90.91 cm³ (95% CI [74.05; 107.77] cm³): 94.04 cm³ (95% CI [67.24; 120.85] cm³) in males versus 87.26 cm³ (95% CI [64.56; 109.97] cm³) in females (*p* = 0.71).

When stratified by age, patients younger than 60 years exhibited a mean T1-enhancing tumor volume of 23.45 cm³ (95% CI [−13.33; 60.23] cm³), compared to 37.92 cm³ (95% CI [26.92; 48.92] cm³) in patients older than 60 years (*p* = 0.38, Fig. 2, D). Similarly, non-enhancing FLAIR hyperintense volumes were 84.30 cm³ (95% CI [14.83; 153.77] cm³) in the younger group versus 91.73 cm³ (95% CI [73.24; 110.22] cm³) in the older group (*p* = 0.78).

Initial T1-contrast-enhanced tumor volume and non-enhancing FLAIR-hyperintense volume showed no correlation with age (Spearman’s *r* = −0.01, *p* = 0.96; *r* = 0.07, *p* = 0.70, respectively) and sex (*r* = −0.20, *p* = 0.26; *r* = −0.07, *p* = 0.70, respectively; see Table 2).

### AR mRNA and protein expression in GBM tissue

**Fig. 3:**
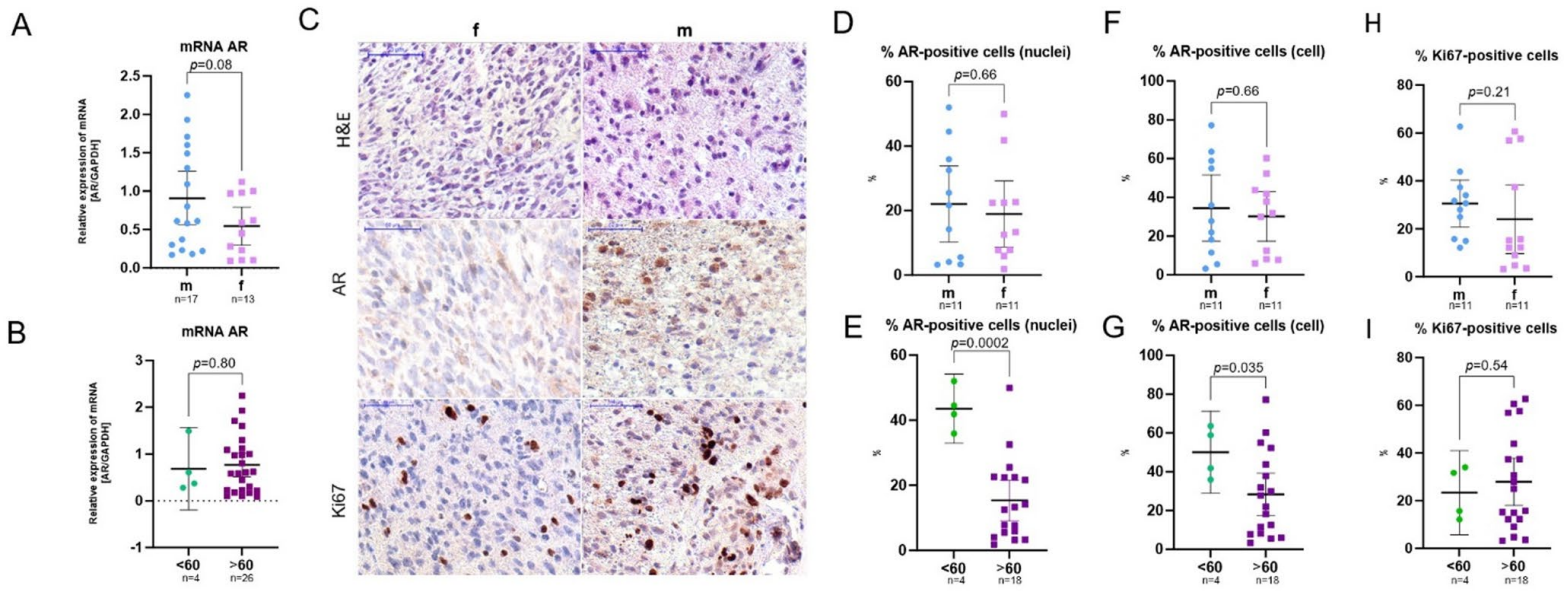
mRNA AR expression in GBM tumor samples depending on sex (**A**) and age (**B**). Representative microphotographs (**C**) show AR and Ki67 protein expression in the GBM tissue (H&E and IHC staining with anti-AR or anti-Ki67 antibodies (immunopositive cells coloured in brown)); scale bar = 50 μm. Scatter dot plots showing mean with 95% CI for % AR- and Ki67- positive cells in GBM tissue depending on sex (**D**, **F**, **H**) and age (**E**, **G**, **I**). m – male, f – female; <60 – patients younger than 60 years, > 60 – patients older than 60 years; AR –androgen receptor; mRNA – messenger ribonucleic acid; GAPDH – Glyceraldehyde-3-phosphate dehydrogenase

Average relative AR mRNA expression in tumor samples was 0.76 (95% CI [0.53; 0.99]), with males showing higher mean expression (0.91 (95% CI [0.56; 1.26]) than females (0.54 (95% CI [0.29; 0.79]) **(**Fig. 3, A). However, this difference was not statistically significant (*p* = 0.08), which is confirmed by medium effect size (Cohen’s d = 0.63) with wide CI (95% CI [–0.13; 1.38]; see Supplementary table).

Patients younger than 60 years had an average AR mRNA expression of 0.69 (95% CI [−0.19; 1.57]) compared to 0.77 (95% CI [0.51; 1.02]) in patients older than 60 years (*p* = 0.80, Fig. 3, B). AR mRNA levels showed tendencies toward inverse correlation with sex (*r* = −0.26, *p* = 0.17) and direct correlation with age (*r* = 0.20, *p* = 0.30; see Table 2). These results should be interpreted cautiously given the very small sample size of the patient subgroup < 60 (*n* = 4).

**Fig. 4:**
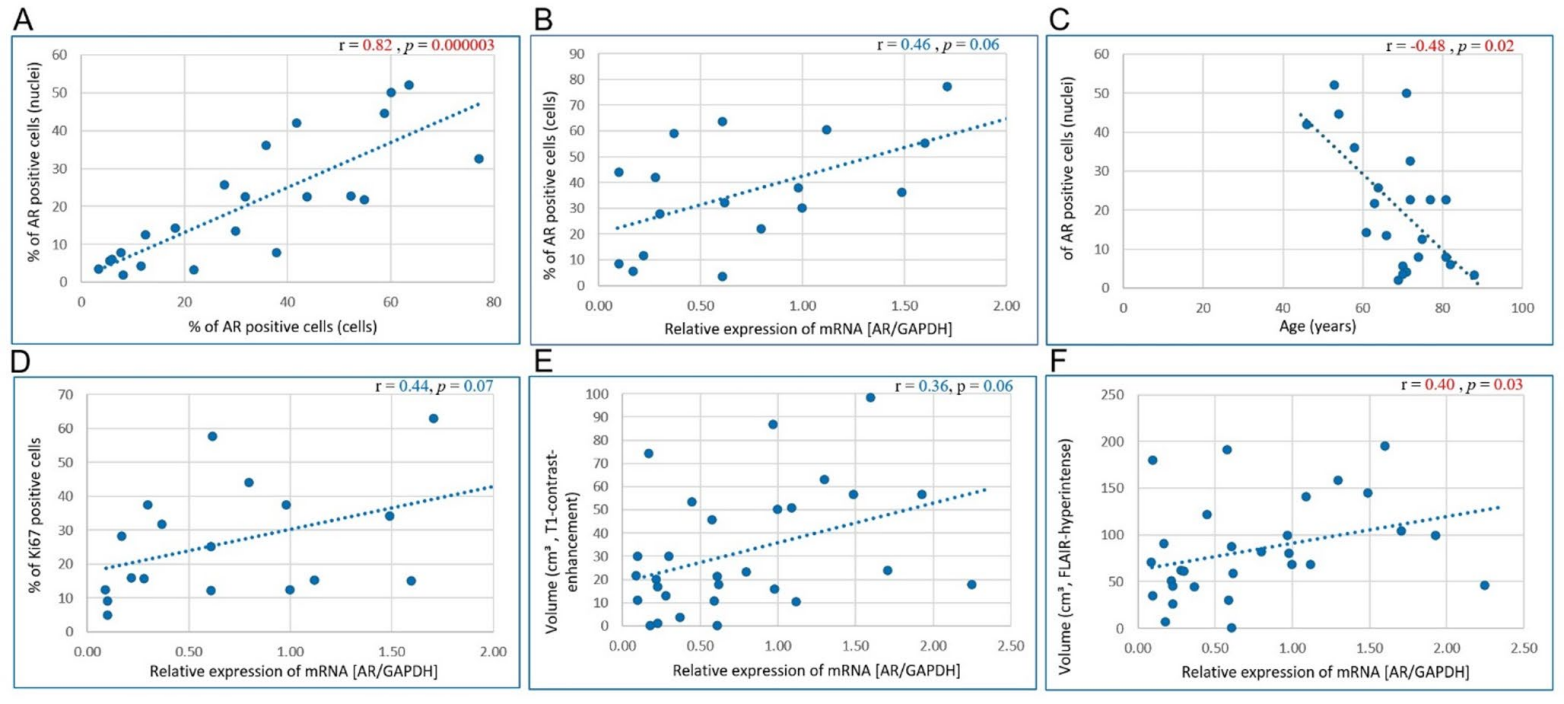
Scatterplots with results of correlation analysis. r *-* Spearman’s rank correlation coefficient; *p*-values < 0.05 (marked in red) are considered significant. AR – androgen receptor; mRNA – messenger ribonucleic acid; GAPDH – Glyceraldehyde-3-phosphate dehydrogenase; FLAIR – fluid-attenuated inversion recovery. Reported *p*-values are exploratory

AR localization was observed both in the nucleus and cytoplasm of tumor cells (Fig. 3, C). The results from two applied approaches for detection of AR-positive tumor cells, based on the DAB-positive nuclei detection (percentage of AR-immunopositive nuclei) or based on the DAB-positive cytoplasm+nuclei (cell) detection (percentage of AR-immunopositive tumor cells) revealed high significant correlation (Spearman’s *r* = 0.82, *p* < 0.0001; Fig. 4, A). Third of the samples (33.3%) had relative amount of AR-positive cells < 10%, and in majority of GBM samples (66.7%) this index exceeded 10%.

The average proportion of cells with AR-positive cell nuclei within the enhancing tumor region was 22.04% (95% CI [10.25; 33.83] %) in male patients and 18.94% (95% CI [8.73; 29.16] %) in female patients (*p* = 0.66, Fig. 3, D). The same pattern was inherent to the amount of AR-positive cells (nuclei+cytoplasm): 34.45% (95% CI [17.33; 51.56] %) in males and 30.20% (95% CI [17.35; 43.06] %) in females (*p* = 0.66, Fig. 3, F).

Mean AR protein expression by tumor cell nuclei was significantly higher in patients under 60 years (43.57% (95% CI [32.96; 54.18] %) compared to those over 60 years (15.36% (95% CI [9.09; 21.64] %; *p* = 0.0002, Fig. 3, E). The magnitude of the effect was exceptionally large (Cohen’s d = 2.37, SE = 0.67, 95% CI [1.06; 3.68]), suggesting a pronounced separation between groups, despite the extremely small sample size of subgroup of patients < 60 (*n* = 4). The same pattern was characteristic for amount of AR-positive cells (nuclei+cytoplasm): 50.07% (95% CI [28.95; 71.19] %) in younger patients and 28.38% (95% CI [17.47; 39.29] %) in older patients (*p* = 0.035, Fig. 3, G). Although the estimated effect size was large (Cohen’s d = 1.04), the wide CI (95% CI [−0.09; 2.17]) reflects considerable uncertainty and does not provide conclusive evidence for a group difference (see Supplementary table).

AR mRNA expression revealed a strong trend for correlation with AR protein expression quantified by IHC staining, as measured by the percentage of AR-immunopositive tumor cells (Spearman’s *r* = 0.46, *p* = 0.06; Fig. 4, B); and no significant correlation with AR-immunopositive tumor nuclei (Spearman’s *r* = 0.26, *p* = 0.31).

Trends suggested no correlation between AR protein expression and sex (*r* =−0.05, *p* = 0.82; *r* =−0.04, *p* = 0.87; see Table 2). However, AR protein expression had inverse correlation with age: non-significant trend for AR-positive cells (nuclei+cytoplasm; *r* = −0.25, *p* = 0.26) and statistically significant correlation (*r* = −0.48, *p* = 0.02; Fig. 4, C) for tumor cell percentage with AR-positive cell nuclei. However, these results should be interpreted with caution given the very small sample size of the subgroup of patients < 60 (*n* = 4).

The average proportion of Ki67-positive cells within the enhancing tumor region did not differ significantly between males and females (*p* = 0.21) and between younger and older patients (*p* = 0.54, Fig. 3, H, I). Nevertheless, the percentage of Ki67-positive tumor cells revealed a strong trend toward association with AR mRNA expression (*r* = 0.44, *p* = 0.07; Fig. 4, D).

Tumor volume showed positive correlation with AR mRNA expression: volume of T1-contrast enhancement regions had a strong trend (*r* = 0.36, *p* = 0.06; Fig. 4, E) and volume of non-enhancing FLAIR-hyperintense regions had a statistically significant correlation (*r* = 0.40, *p* = 0.03; Fig. 4, F). At the same time, tumor volume indices showed no association with AR protein expression measured as the percentage of AR-immunopositive tumor nuclei/cells (*r* = −0.14, *p* = 0.55; *r* = −0.16, *p* = 0.48; *r* = 0.08, *p* = 0.74; *r* = 0.14, *p* = 0.54, respectively; see Table 2).

It should be noted that because multiple endpoints/correlations were tested, the *p*-values ​​reported are exploratory.

**Table 2:**
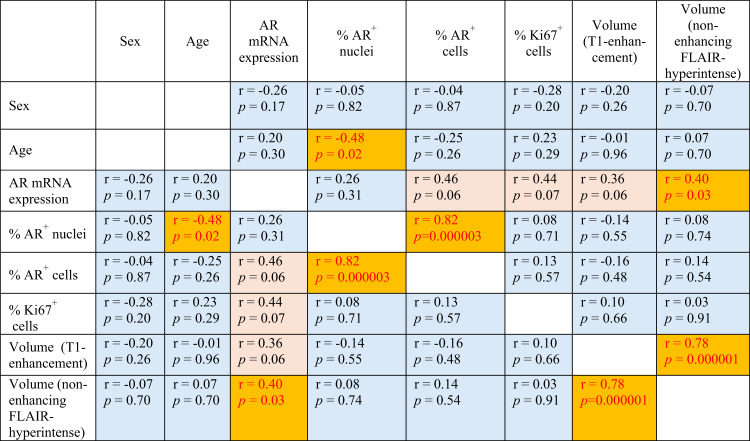
Correlation analysis results

r - Spearman’s rank correlation coefficient; *p*-values < 0.05 (marked in red) are considered significant; *p* = (0.05–0.10) marked in pink; *p* = (0.10–0.97) marked in blue. AR – androgen receptor; mRNA – messenger ribonucleic acid; FLAIR – fluid-attenuated inversion recovery. Reported *p*-values are exploratory

### Exploratory multivariable linear regression models

Multivariable linear regression models were fitted to assess independent associations of AR expression with log-transformed tumor volumes (T1-contrast-enhancing and FLAIR-hyperintense regions), adjusting for age, sex, MGMT methylation status and proliferation (% of Ki67 positive cells).

**Fig. 5:**
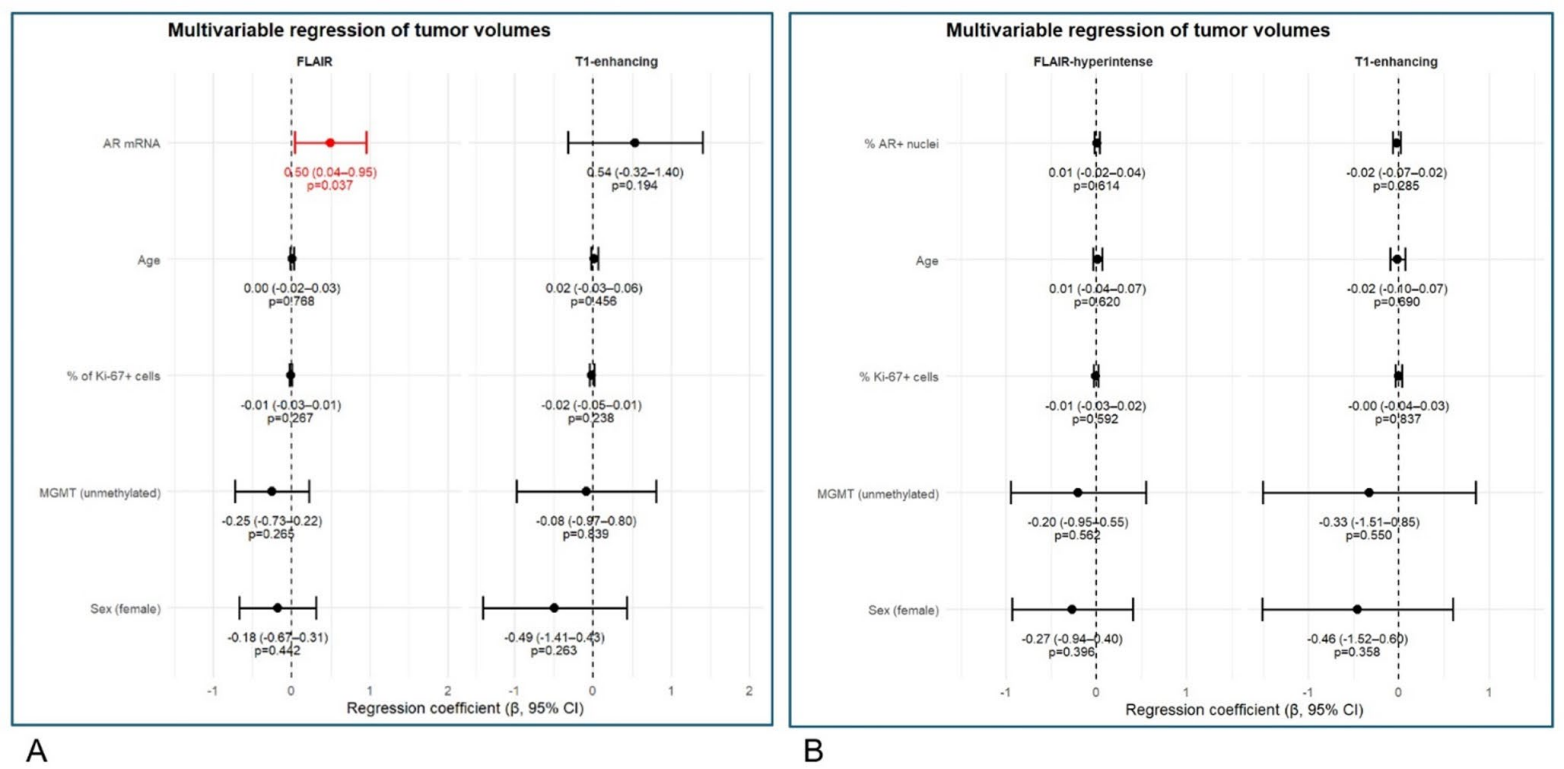
Multivariable regression analysis of tumor volume compartments. Forest plots showing regression coefficients (β) with 95% confidence intervals for associations between AR mRNA expression (**A**), % AR positive tumor cells (AR+ nuclei) (**B**) and Ki67 proliferation index, age, sex, and MGMT promoter methylation with log-transformed FLAIR-hyperintense (left) and T1 contrast-enhancing (right) tumor volumes. Estimates are adjusted for all covariates included in the model. Red markers indicate statistically significant associations (*p* < 0.05), while black markers indicate non-significant results. Labels display β coefficients with 95% confidence intervals and corresponding *p*-values. The dashed vertical line represents no effect (β = 0)

AR mRNA expression was independently associated with larger FLAIR hyperintense volume (β = 0.50, 95% CI: 0.04–0.95, *p* = 0.037), but not T1-enhancing volume (β = 0.54, 95% CI: − 0.32–1.40, *p* = 0.196). No other covariates showed statistically significant independent associations with either volume measure (Fig. 5, A).

At the same time, AR protein expression, assessed as the percentage of AR-positive tumor cells (nuclei)), showed a weak trend toward association with infiltrative FLAIR-hyperintense volume (β = 0.01, 95% CI: −0.02–0.04, *p* = 0.614), but not clear relationship with contrast-enhancing volume after multivariable adjustment (Fig. 5, B). No other covariates (age, proliferation index, MGMT status, sex) showed statistically significant independent assotiations with either volume measure. The effect sizes are small (β < 0.1), suggesting limited explanatory power of these predictors for tumor volume variation.

Overall, these findings suggest a potential link between AR transcriptional activity and tumor-associated infiltrative tumor growth reflected by FLAIR-hyperintense signal, which is not recapitulated at the protein level.

## Discussion

In this study, we investigated the relationship between AR expression – at both the mRNA and protein levels – and tumor volume in the enhancing region of GBM tissue, with additional analysis of associations with patient sex and age. Although AR signaling is best known for its role in prostate cancer, accumulating evidence indicates that it may also contribute to GBM biology. Experimental and clinical data suggest that AR activity could influence tumor development, progression, and therapy resistance, yet its exact role in GBM remains incompletely understood.

Epidemiological observations point toward a potential sex-specific involvement of AR in GBM: the incidence rate of GBM in men is approximately 1.6-fold higher than in women [7, 12, 13], and glioma patients have been reported to exhibit elevated serum testosterone levels compared to non-cancer controls [50]. On a genomic level, AR amplification is detected in 27–38% of GBM cases in both sexes, and the constitutively active splice variant AR-V7/AR3 – capable of ligand-independent signaling – is present in about 30% of GBM, where it contributes to tumor growth [18].

AR upregulation in GBM tissue has been documented in several studies, with higher expression linked to advanced WHO grades [7, 15, 24, 28, 29, 50]. Immunohistochemical analyses reveal AR overexpression in up to 96.3% of grade 4 gliomas, often with particularly intense staining in perivascular regions [29, 50]. *In vitro*, AR activation, either androgen-dependent or independent, promotes GBM cell proliferation, migration, and invasion, whereas AR silencing induces cell death [21, 22, 29, 31]. Clinically, high AR activity seems to correlate with poorer overall survival [22].

Preclinical models further support a functional role for AR in GBM progression. Pharmacological inhibition with enzalutamide reduced subcutaneous glioma xenograft volume by 72% in mice [18]. Moreover, AR antagonists have been shown to increase radiosensitivity, potentially via modulation of TGF-β/LIF/STAT3 signaling [32, 40], and male mice with GBM responded better to combined radio- and chemotherapy. AR antagonists alone or in combination with temozolomide demonstrated the efficacy in extending the lifespan of mice with intracranial human GBM, suggesting a promising approach to enhance patient outcomes [20]. These findings position AR signaling as a possible therapeutic target in GBM [18–20, 23, 41].

Our analysis focused on the enhancing tumor region, following the previous findings [30], reported higher AR protein expression in this region than in the peritumoral area or tumor core. IHC staining to AR was performed using AR antibody (441): sc-7305 (Santa Cruz Biotechnology) detecting AR protein expression both in cytoplasm and nucleus of tumor cells [51]; similar expression patterns are also shown in several publications [23, 30, 41, 50].

According to databases, there are at least 7–8 transcript variants for AR in humans [52] and > 20 annotated transcript variants, of which about 8–12 are predicted to be protein-coding isoforms [53]. AR antibody (441): sc-7305 is a monoclonal antibody raised against amino acids 299–315 of the human AR [51] – region, which lies in the DNA-binding domain (DBD) or immediately adjacent region (depending on exact AR isoform), which is present in full-length AR (AR-FL) and also in many splice variants that retain the DBD. Because the epitope is fairly upstream of the ligand-binding domain (LBD), any isoform that retains the DBD and retains that stretch (aa 299–315) will likely be recognized by this antibody. So, SC-7305 will detect full-length AR, it does not specifically discriminate AR-V7 splice variant [54].

Located primarily in the cytoplasm and nucleus of target cells, AR undergoes a conformational change upon binding to ligands, allowing translocation to the nucleus where AR regulates gene expression. It is nuclear localization that is essential for function AR as a transcription factor, influencing biological processes, including cell growth, differentiation, and apoptosis [17, 51]. Given this information, we included in the analysis both tumor cells with positively stained nuclei (nuclei detection) and positively stained cytoplasm+nuclei (cell detection). As these two indices showed high significant correlation (*r* = 0.82, *p* < 0.0001), we further focus on positive nuclei detection, that is, with nuclear localization of the AR.

In our cohort, AR mRNA and protein expression levels varied substantially among patients but showed a trend for positive correlation (*r* = 0.26, *p* = 0.31; *r* = 0.46, *p* = 0.06, respectively for positive nuclei or cell detection). This trend is consistent with results from Fariña-Jerónimo et al. [22], who found a similar positive association (*r* = 0.56, *p* < 0.0001) using The Cancer Genome Atlas (TCGA)-derived data. Although mean AR expression was higher in males than females, the difference was not statistically significant, in agreement with prior reports [22, 30, 40].

Tumor volume demonstrated direct correlation with AR mRNA levels: a strong trend for T1-contrast enhancement regions (*r* = 0.36, *p* = 0.06) and significant correlation for non-enhancing FLAIR-hyperintense regions (*r* = 0.40, *p* = 0.03); while the association with AR protein expression was weaker and non-significant.

Evaluating both tumor volume parameters allows for a more comprehensive assessment of tumor burden and progression dynamics: T1-contrast-enhancing volume quantifies the actively proliferating tumor core with blood-brain barrier disruption, while the non-enhancing FLAIR-hyperintense volume represents infiltrative tumor tissue surrounding the core. Thus the identified trend of a stable association of tumor volume indices with AR mRNA expression indicates potential role of AR-dependent downstream signaling pathways in GBM progression.

This assumption is also supported by the association we found between the proliferative index of GBM tissue (percentage of Ki67-positive tumor cells) and AR mRNA expression (*r* = 0.44, *p* = 0.07), consistent with prior observations [30].

No statistically significant differences in tumor volume or AR mRNA expression were observed based on sex or age, though trends indicated higher AR mRNA expression in older patients and males.

At the same time, AR protein expression in GBM tissue showed no association with patient sex but revealed an age-related decline. Mean nuclear AR expression was significantly higher in patients under 60 years compared to older patients (*p* = 0.0002), demonstrating inverse correlation with age (*r* = −0.48, *p* = 0.02). This stratification (age < 60 vs. ≥60 years) was tested in accordance with established clinical research and WHO classification criteria [47–49]. Nevertherless, due to the very small subgroup of patients under 60 years (*n* = 4) in our patient cohort, this finding is rather as hypothesis-generating and need further validation on appropriate patient cohort.

After applying the exploratory multivariable linear regression models and adjustment for age, sex, proliferative index (% of Ki67-positive cells), and MGMT status, higher AR mRNA expression was independently associated with greater infiltrative tumor burden (FLAIR-hyperintense volume), but not with contrast-enhancing tumor size, while AR protein and Ki67 showed no significant associations — suggesting AR signaling is linked to invasive rather than proliferative GBM behavior. These results should be interpreted cautiously given the limited sample size, but the consistent pattern across models supports biological plausibility and point toward an invasion-associated role of AR signaling.

Association of AR transcriptional activity and tumor-associated infiltrative growth reflected by FLAIR-hyperintense signal is not recapitulated at the protein level.

Thus, we obtained a discrepancy between AR mRNA and protein expression. This may be due to several reasons.

Firstly, this discordance is consistent with extensive evidence that mRNA abundance does not directly predict protein levels because gene expression is controlled at multiple regulatory points beyond transcription, including mRNA processing, translation, and post-translational modifications [55–57]. Protein levels are influenced by translation efficiency, alternative splicing, mRNA stability, and regulated protein degradation, all of which can vary with age, stress responses, and tumour context. Particularly, RNA-binding proteins (YTHDF3 and G3BP1), miRNAs, initiation/elongation factors and modification of AR mRNA (e.g., N₆-methyladenosine, m6A) can modulate its translation independently of transcription, and post-translational processes can target AR for degradation, leading to reduced protein despite higher mRNA [57]. Such buffering and post-transcriptional control mechanisms are widely observed across tissues and species and likely contribute to the observed AR mRNA–protein discordance in our GBM cohort.

Second, alternative splicing or AR-antibody epitope specificity cannot be ruled out. AR transcripts are known to undergo complex alternative splicing, producing multiple isoforms with different coding potential and stability. Age-associated changes in splicing regulation are well documented in cancer, and splicing shifts can generate mRNA isoforms that are inefficiently translated or targeted for decay [58].

These discrepancies might also account for the relatively low staining intensity observed in the IHC for GBM tissue, which appears markedly stronger when examined in sections of prostatic carcinoma.

Third, protein levels can be degraded via ubiquitin–proteasome system, autophagy, regulated turnover (e.g., age-dependent changes in proteostasis); phosphorylation, acetylation, ubiquitination, and other modifications can affect AR’s stability and activity. This can lead to decreased protein levels even when mRNA is high. Tumour microenvironment, hypoxia, inflammation, and metabolic stress can alter the efficiency of translation and protein stability independently of AR mRNA levels. Regulatory pathways activated in tumors may upregulate AR transcription as a compensatory response, while simultaneously increasing degradation or translational repression of AR protein.

Published data on the relationship between AR expression and patient age in GBM remain inconsistent. A TCGA-based analysis by Zalcman et al. [18] reported a weak but statistically significant positive correlation between AR mRNA levels and age in GBM samples (Spearman *r* = 0.11, *p* = 0.01), while subsequent database-wide studies did not confirm a clear age dependence [24, 30]. Conversely, smaller IHC studies have suggested variable or even opposite trends, likely reflecting differences in detection methods (mRNA vs. protein), cohort size, and tumor heterogeneity. Such methodological and biological variability may explain the mixed findings across studies, underscoring the need for standardized multimodal analyses in larger cohorts.

Taken together, our results indicate that AR mRNA expression in GBM tissue may be associated with tumor volume, supporting the hypothesis that AR potentially contributes to GBM progression. These findings are preliminary, given the modest sample size and the limited statistical power, which resulted in borderline significant results, that are mostly exploratory trends rather than definitive conclusions. Also we cannot exclude the possible confounders such as age, sex, and corticosteroid use. As mentioned above, information on prior steroid administration was not consistently available for all patients, as a subset of cases was referred from external institutions. Ongoing patient recruitment will allow for more robust statistical analyses and correlate with outcome data.

Our results are nonetheless consistent with the observation that the actively proliferating tumor regions exhibit the highest AR expression [30], and with experimental data suggesting that AR signaling promotes GBM aggressiveness [21].

Future studies should address not only AR expression but also the functional activation status of the receptor and its downstream transcriptional targets, to clarify the role of androgen signaling in GBM pathophysiology [22]. Pathways regulated by AR include those involved in growth factor signaling (EGFR, PI3K), cell cycle control (CDK4/6, cyclin D1), and extracellular matrix remodeling (MMPs) [17], all of which could contribute to tumor aggressiveness.

Future studies should also address the role of the androgen receptor (AR) in GBM radioresistance and its regulation following irradiation. Radiotherapy is a cornerstone of GBM treatment; however, its effectiveness is often limited by intrinsic resistance mechanisms of tumor cells [37]. Emerging evidence suggests that AR expression may contribute to radioresistance in GBM. High AR levels have been associated with reduced sensitivity to radiation, whereas pharmacological inhibition of AR using antagonists such as enzalutamide or seviteronel increased radiosensitivity in experimental GBM models [40, 41].

A better understanding of AR-downstream mechanisms and elucidating the role of AR in the GBM radiation response may provide a biological basis for sex-related differences in disease outcome and uncover opportunities to integrate AR-targeted therapies into GBM treatment strategies.

## Conclusion

AR expression is consistently detectable in GBM tissue and shows a trend toward association at the transcriptional level with non-contrast-enhancing tumor volume, suggesting a potential role in GBM biology and warranting further investigation in larger studies.

## Supplementary Information

Below is the link to the electronic supplementary material.

Supplementary Material 1: Figure S1: Melting curves for qPCR using AR and GAPDH primers (Bio-Rad CFX Manager 3.1, Bio-Rad Laboratories)

Supplementary Material 2

## Data Availability

No datasets were generated or analysed during the current study.

## Abbreviations

AR: Androgen receptor
BLAST: Basic Local Alignment Search Tool
cDNA: Complementary deoxyribonucleic acid
DAB: Diaminobenzidine
DBD: DNA-binding domain
EGFR: Epidermal Growth Factor Receptor
FFPE: Formalin fixed & paraffin-embedding
FLAIR: Fluid-Attenuated Inversion Recovery
GAPDH: Glyceraldehyde-3-phosphate dehydrogenase
GBM: Glioblastoma
H&E: Hematoxylin-eosin
IDH: Isocitrate dehydrogenase
IHC: Immunohistochemistry
LBD: Ligand-binding domain
MGMT: O^6^-methylguanine-DNA methyltransferase
MRI: Magnetic resonance imaging
mRNA: Messenger ribonucleic acid
OD: Optical density
qPCR: Quantitative real-time polymerase chain reaction
TCGA: The Cancer Genome Atlas
TTF: Tumor treating fields
WHO: World Health Organization
