# Supplementary figures and images for: Androgen receptor expression in glioblastoma: molecular profiling and association with tumor burden
Source: Mol Biol Rep. 2026 Mar 24;53(1):531. doi: 10.1007/s11033-026-11673-6 (PMC13013117; doi:10.1007/s11033-026-11673-6)

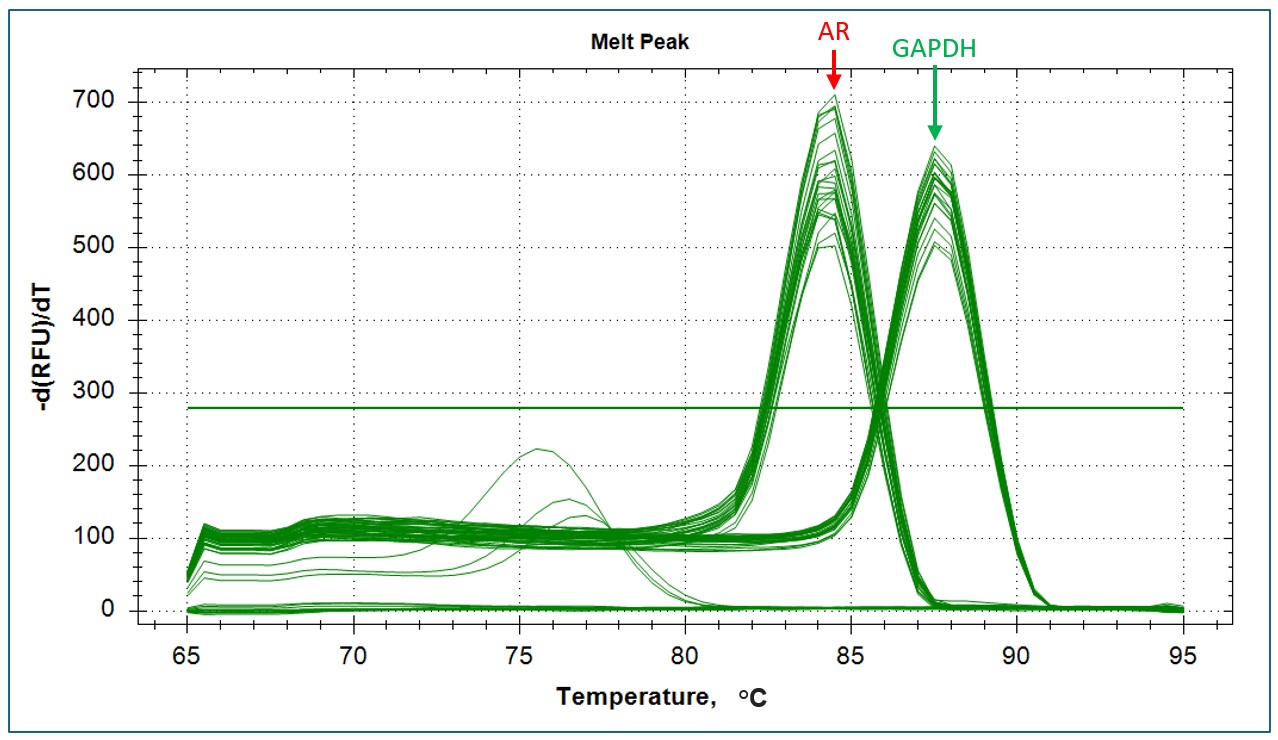

Supplement: Supplementary file 1 — Supplementary Material 1: Figure S1: Melting curves for qPCR using AR and GAPDH primers (Bio-Rad CFX Manager 3.1, Bio-Rad Laboratories) [file 11033_2026_11673_MOESM1_ESM.jpg]
