# Supplementary material for: Androgen receptor expression in glioblastoma: molecular profiling and association with tumor burden
Source: Mol Biol Rep. 2026 Mar 24;53(1):531. doi: 10.1007/s11033-026-11673-6 (PMC13013117; doi:10.1007/s11033-026-11673-6)
Supplement: Supplementary file 2 — Supplementary Material 2 [file 11033_2026_11673_MOESM2_ESM.docx]

**Supplementary table.** Results of the pairwise (*t-test with Welch’s correction*) comparisons between groups.

| Index | Pairwise comparison  (*t-test with Welch’s correction)* | *p*-value | Effect size (*Cohen’s d*) | Standart error  (SE) | 95 % confidence intervals (CI) | |
| --- | --- | --- | --- | --- | --- | --- |
|  |  |  |  |  | lower CI | upper CI |
| Tumor volume  (T1-contrast-enhanced MRI),  cm³ | Sex:  m (n=18) *vs.* f (n=16) | 0.21 | 0.18 | 0.35 | –0.50 | 0.87 |
|  | Age:  >60 (n=30) *vs.* <60 (n=4) | 0.38 | –0.45 | 0.54 | –1.51 | 0.60 |
| Tumor volume  (non-enhancing FLAIR-hyperintense MRI),  cm³ | Sex:  m (n=18) *vs.* f (n=16) | 0.71 | 0.13 | 0.35 | –0.56 | 0.81 |
|  | Age:  >60 (n=30) *vs.* <60 (n=4) | 0.78 | –0.14 | 0.53 | –1.19 | 0.90 |
| AR mRNA expression | Sex:  m (n=17) *vs.* f (n=13) | 0.08 | 0.63 | 0.39 | –0.13 | 1.38 |
|  | Age:  >60 (n=26) *vs.* <60 (n=4) | 0.80 | –0.13 | 0.54 | –1.19 | 0.92 |
| AR-positive cells (nuclei),  % | Sex:  m (n=11) *vs.* f (n=11) | 0.66 | 0.19 | 0.43 | –0.65 | 1.03 |
|  | Age:  >60 (n=26) *vs.* <60 (n=4) | 0.0002 | 2.37 | 0.67 | 1.06 | 3.68 |
| AR-positive cells (cells),  % | Sex:  m (n=11) *vs.* f (n=11) | 0.66 | 0.19 | 0.43 | –0.65 | 1.03 |
|  | Age:  >60 (n=26) *vs.* <60 (n=4) | 0.035 | 1.04 | 0.58 | –0.09 | 2.17 |
| Ki67-positive cells,  % | Sex:  m (n=11) *vs.* f (n=11) | 0.21 | 0.34 | 0.42 | –0.48 | 1.17 |
|  | Age:  >60 (n=26) *vs.* <60 (n=4) | 0.54 | –0.24 | 0.55 | –1.32 | 0.84 |

*p*-values < 0.05 are considered significant (marked in red). AR – androgen receptor; mRNA – messenger ribonucleic acid; m – male, f – female; <60 – patients younger than 60 years, >60 – patients older than 60 years; MRI – magnetic resonance imaging; FLAIR – fluid-attenuated inversion recovery.
